# Muscarinic receptors modulate Nerve Growth Factor production in rat Schwann-like adipose-derived stem cells and in Schwann cells

**DOI:** 10.1038/s41598-020-63645-w

**Published:** 2020-04-28

**Authors:** R. Piovesana, A. Faroni, M. Taggi, A. Matera, M. Soligo, R. Canipari, L. Manni, A. J. Reid, A. M. Tata

**Affiliations:** 1 Department of Biology and Biotechnologies “Charles Darwin”, Sapienza, University of Rome, Rome, Italy; 2 Blond McIndoe Laboratories, Division of Cell Matrix Biology and Regenerative Medicine, School of Biological Sciences, Faculty of Biology, Medicine and Health, The University of Manchester, Manchester Academic Health Science Centre, Manchester, M13 9PT UK; 3 DAHFMO, Unit of Histology and Medical Embryology, Sapienza, University of Rome, Rome, Italy; 4 Institute of Translational Pharmacology, National Research Council of Italy (CNR), Rome, Italy; 5 Department of Plastic Surgery & Burns, Wythenshawe Hospital, Manchester University NHS Foundation Trust, Manchester Academic Health Science Centre, Manchester, UK; 6 Research Centre of Neurobiology “Daniel Bovet”, Sapienza, University of Rome, Rome, Italy

**Keywords:** Neuroscience, Glial biology

## Abstract

Regenerative capability of the peripheral nervous system after injury is enhanced by Schwann cells (SCs) producing several growth factors. The clinical use of SCs in nerve regeneration strategies is hindered by the necessity of removing a healthy nerve to obtain the therapeutic cells. Adipose-derived stem cells (ASCs) can be chemically differentiated towards a SC-like phenotype (dASCs), and represent a promising alternative to SCs. Their physiology can be further modulated pharmacologically by targeting receptors for neurotransmitters such as acetylcholine (ACh). In this study, we compare the ability of rat dASCs and native SCs to produce NGF *in vitro*. We also evaluate the ability of muscarinic receptors, in particular the M2 subtype, to modulate NGF production and maturation from the precursor (proNGF) to the mature (mNGF) form. For the first time, we demonstrate that dASCs produce higher basal levels of proNGF and mature NGF compared to SCs. Moreover, muscarinic receptor activation, and in particular M2 subtype stimulation, modulates NGF production and maturation in both SCs and dASCs. Indeed, both cell types express both proNGF A and B isoforms, as well as mNGF. After M2 receptor stimulation, proNGF-B (25 kDa), which is involved in apoptotic processes, is strongly reduced at transcript and protein level. Thus, we demonstrate that dASCs possess a stronger neurotrophic potential compared to SCs. ACh, via M2 muscarinic receptors, contributes to the modulation and maturation of NGF, improving the regenerative properties of dASCs.

## Introduction

Neurotrophins are a group of proteins that specifically stimulate neuronal activity during development, adult life and injury, supporting neuron survival, neurite outgrowth and controlling neuron and glial cell differentiation^1^.

Nerve growth factor (NGF) has particular relevance for the survival and functional activity of sensory and sympathetic neurons^2,3^. NGF is synthesised as a precursor (proNGF) which is proteolytically cleaved to produce mature NGF (mNGF)^3,4^. In particular, two different isoforms of proNGF are described: proNGF-A (32 kDa) and proNGF-B (25 kDa)^5^. There are consistent clues that proNGF-A isoform may act as a modulator of cell survival^6^, whereas proNGF-B isoform acts as an apoptotic signal^7^. proNGF undergoes post-translational intracellular and extracellular processing at both amino- and carboxyl-terminal ends, thanks to the action of plasmin cleavage, to produce the mNGF form of 13.2kDa^4^. Both proNGF and mNGF can be secreted and have active functional roles^5,7,8^. In healthy peripheral nerves, NGF is produced by the target organs and retrogradely transported to the neuronal soma^1^ binding two distinct receptors: the high-affinity Tropomyosin receptor kinase A (TrkA) and the low-affinity p75NTR^9,10^. After nerve injury, axons are dissociated from the source of neurotrophic support, and the Schwann cells (SCs) lose their contact with the axons before acquiring the *repair* phenotype. At this stage, nerve regeneration is promoted by removal of myelin debris and by production of neurotrophic factors including NGF, by *repair* SCs^11–13^.

Despite the presence of *repair* SCs, conferring intrinsic regenerative potential to the injured peripheral nervous system (PNS), full functional recovery is always incomplete. This has driven experimental work on the use of cellular therapy as a potential clinical intervention to promote the recovery of injured peripheral nerve; however, SCs therapeutic potential is limited by the need to sacrifice a healthy nerve and by their slow *in vitro* proliferative rate^14^.

Other cell types, such as adipose-derived stem cells (ASCs), possess the ability to differentiate towards SCs phenotype (SC-like, dASCs) when exposed to specific growth factors (glial growth factor, GGF; Platelet-Derived Growth Factor, PDGF; Basic Fibroblast Growth Factor, bFGF; Forskolin, Fsk)^15,16^. The ease of ASCs harvesting and the rapid differentiation in SCs phenotype make Schwann–like (dASCs) an excellent candidate to further investigate for their translational potential in peripheral nerve injury.

In recent years, promising roles have emerged for neurotransmitters^17–20^, including ACh^21–25^, in regulating important processes in glial cells of the central (CNS) and PNS. Indeed, in the PNS muscarinic receptors are present on both neurons and non-neuronal cells of the sensory ganglia^26^. Furthermore, in the CNS, muscarinic receptors are developmentally regulated in oligodendrocytes^27^. This evidence suggests an important role for ACh as mediator of neuron-glia cross-talk in both CNS and PNS^28^. Rat SCs express distinct muscarinic receptor subtypes, with greater expression of M2 subtype^21^. M2 selective activation with agonist Arecaidine Propargyl Ester (APE) inhibits SCs proliferation^22^, upregulating promyelinating genes (e.g. Sox10 and EGR2) and myelin proteins (e.g. P0 and MBP)^23^. dASCs express functional receptors for several neurotransmitters such as GABA, ATP^29–31^ and all muscarinic receptor subtypes^32,33^. In dASCs, M2 receptor activation produces a reversible decrease of cell proliferation, reduces migration and enhances dASCs differentiation as shown by improved spindle shaped morphology accompanied by early growth factor 2 (EGR2) upregulation^33^. dASCs produce neurotrophic factors, such as BDNF (Brain-derived neurotrophic factor, BDNF) and NGF, which are important for their neurotrophic effects as demonstrated in animal models of peripheral nerve regeneration^34,35^.

In this work, we have evaluated the ability of muscarinic receptors to modulate NGF production and release in rat dASCs and SCs. For the first time, we demonstrate that dASCs produce and release higher levels of proNGF and mNGF than SCs. We have also analysed the effects of non-selective muscarinic agonist stimulation (muscarine) and M2 selective agonist stimulation (APE) on NGF production and maturation in both dASCs and native SCs. Our results indicate that muscarinic receptor activation triggers NGF production both in SCs and in dASCs. These results may contribute to define a new pharmacological target, improving the neurotrophic potential of dASCs towards new therapeutic approaches for peripheral nerve regeneration.

## Results

### Cholinergic modulation of NGF expression

**Figure 1:**
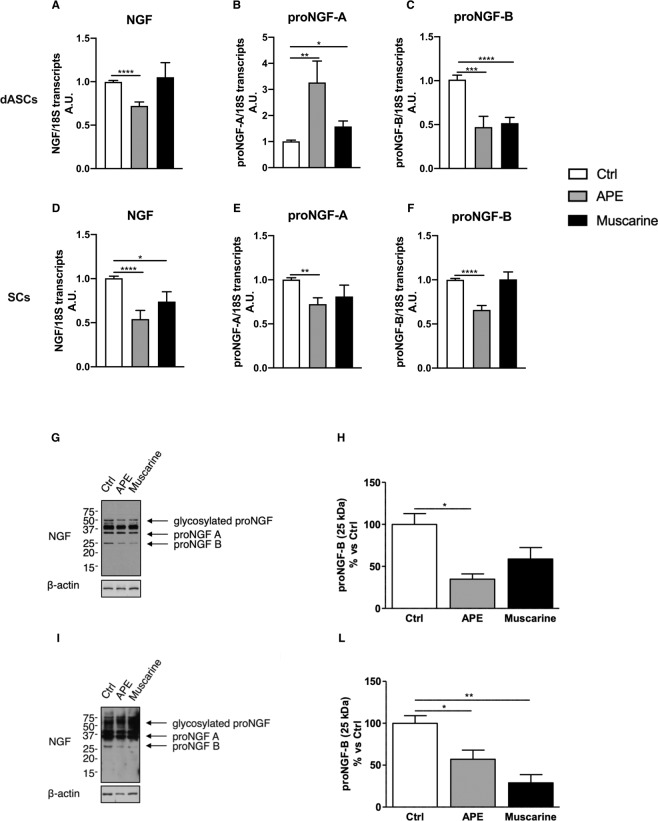
Expression of Nerve Growth Factor in dASCs and SCs after 24 h of cholinergic treatments. (**A**, **D**) NGF gene expression levels were significantly decreased after 24 h of APE treatment both in dASCs (fold change: 0.7213 ± 0.045, ****P < 0.0001; n = 4) and SCs (fold change: 0.5425 ± 0.097, ****P < 0.0001; n = 4), whereas muscarine is able to decrease the NGF levels only in SCs (fold change: 0.7395 ± 0.11, *P < 0.05; n = 4). After APE and muscarine exposures a proNGF-A consistent upregulation was observed in dASCs (**B**, APE fold change: 3.270 ± 0.82, **P = 0.0048; muscarine fold change: 1.583 ± 0.21; *P < 0.05; n = 4) while a significant decrease of proNGF-A was observed in SCs after APE treatment (**E**, fold change: 0.7239 ± 0.072, **P  = 0.0012; n = 4). APE treatment downregulated proNGF-B isoform in both cell types (**C**, fold change: 0.4724 ± 0.12, ***P = 0.0007; **F**, fold change: 0.6589 ± 0.050, ****P < 0.0001; n = 4). A significant downregulation was observed in dASCs after muscarine treatment (**C**, fold change: 0.5168 ± 0.065, ****P < 0.0001; n = 4) whereas any effect was observed in SCs (**F**). (**G**, **I**) Western blotting showing expression of different proNGF isoforms. After APE exposure, proNGF-B protein levels strongly decreased in both cell types (**H**, **L**) (34.78 ± 6.32% vs Ctrl, *P < 0.05; 57.05 ± 10.87% vs Ctrl, *P < 0.05; n = 3). After muscarine treatment there was a decrease of proNGF-B protein expression but it was significant only in SCs (**L**, 28.94 ± 9.72% vs Ctrl, **P = 0.0045; n = 3). For quantification of western blot analysis, levels of NGF expression were normalised for the housekeeping protein β-actin, used as a loading control, and expressed as percentage vs non-treated controls ± SEM.

Firstly, we investigated the ability of muscarinic agonists to modulate NGF expression after 24 h of treatment. NGF transcript levels were significantly decreased following APE treatments in both dASCs and SCs (Fig. 1A,D), compared to untreated controls, whereas muscarine was able to reduce NGF gene expression only in SCs (Fig. 1D).

Gene expression analysis showed that APE exposure was able to upregulate proNGF-A in dASCs compared to untreated controls (Fig. 1B), while a significant decrease was observed in SCs (Fig. 1E). On the other hand, the non-selective agonist muscarine upregulated proNGF-A transcript only in dASCs (Fig. 1B), but its protein levels remained still unmodified (Fig. 1G).

proNGF-B isoform expression was significantly downregulated after APE treatment (Fig. 1C, F), whereas muscarine treatment was able to significantly decrease its expression only in dASCs (Fig. 1C).

Following 48 h of exposure to 100 μM of APE or muscarine, rat dASCs did not display any variation of proNGF-A protein but showed lower levels of 25 kDa proNGF-B, compared to the untreated cells (Fig. 1G,H).

In SCs, the protein levels of proNGF-A remained unmodified whereas the 25 kDa proNGF-B isoform was significantly downregulated following both treatments (Fig. 1I, L).

**Figure 2:**
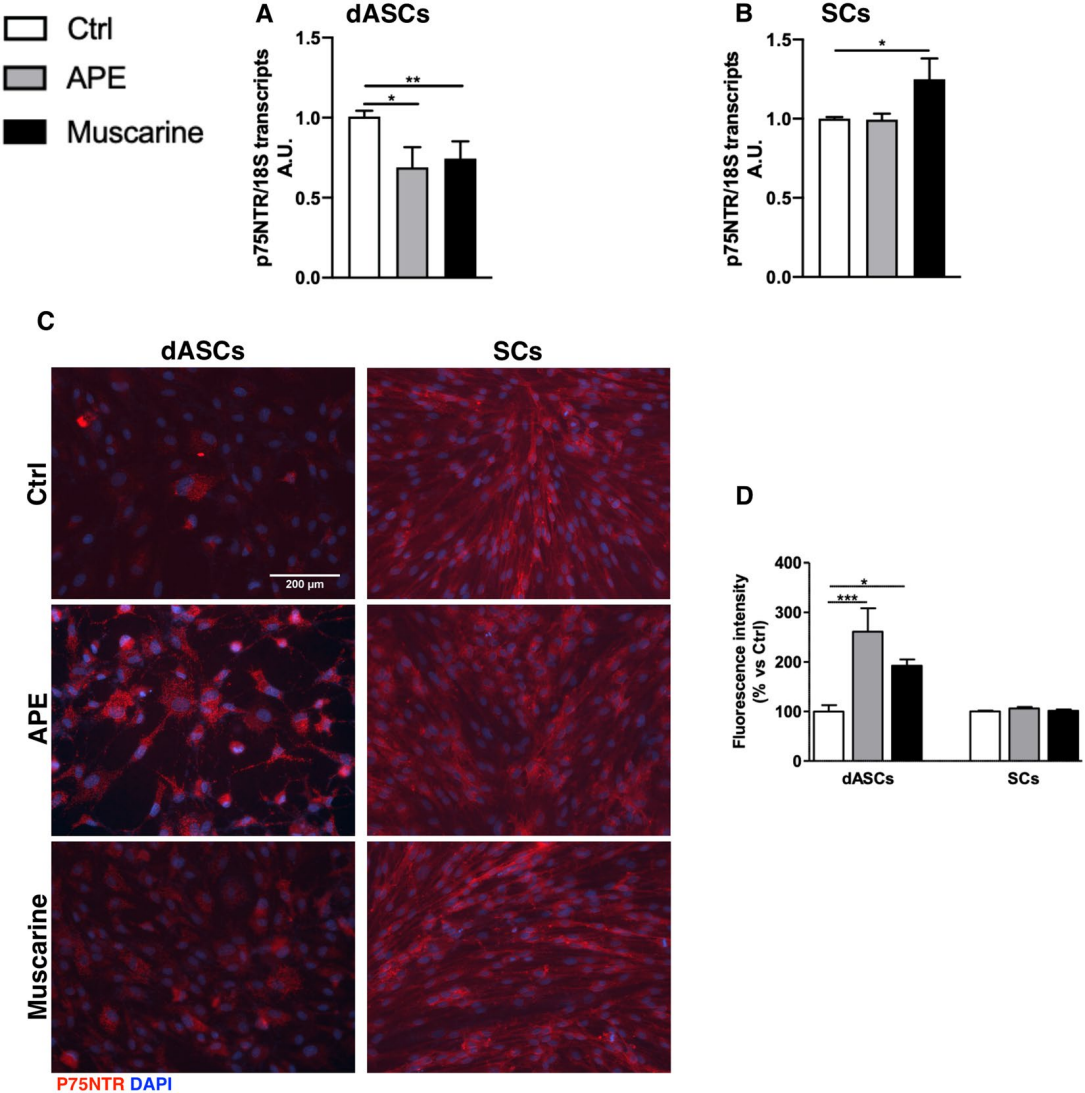
p75NTR expression in dASCs and SCs. Low-affinity nerve growth factor receptor (p75NTR) gene expression levels were significantly decreased in dASCs after both drugs exposure (**A**; APE fold change: 0.6896 ± 0.126, *P = 0.0197; muscarine fold change: 0.7443 ± 0.108, **P = 0.0085; n = 4). p75NTR was upregulated after muscarine treatment in SCs (**B**, fold change: 1.250 ± 0.12, *P < 0.05; n = 4). (**C**) Immunocytochemistry analysis of p75NTR protein in dASCs and SCs (scale bar = 200 μm). (**D**) Analysis of fluorescence intensity of p75NTR immunostaining. APE and muscarine treatments increased p75NTR expression in dASCs (APE: 261.3 ± 47.07% vs Ctrl, ***P < 0.001; muscarine: 192.3 ± 12.84% vs Ctrl, *P < 0.05).

ProNGF isoforms typically bind low-affinity nerve growth factor receptor p75NTR^7,36^. qPCR analyses demonstrated that p75NTR was significantly decreased in dASCs after both muscarinic agonists treatments (Fig. 2A), but it was upregulated after muscarine treatment in SCs (Fig. 2B). Immunocytochemistry analysis confirmed the expression of p75NTR receptor in both cell types (Fig. 2C), however the expression of the receptor was not altered after muscarinic agonists treatments in SCs cells (Fig. 2C). In dASCs, the expression of p75NTR receptor was increased upon APE and muscarine treatment (Fig. 2C). Densitometric analysis of p75NTR immunostaining demonstrated an increased of p75NTR expression in dASCs upon both muscarinic agonist treatments in dASCs (Fig. 2D). Conversely, p75NTR expression was unaltered in SCs upon muscarinic challenge (Fig. 2C, D).

### dASCs produce and release higher basal levels of NGF

Several studies described how dASCs produce neurotrophic factors increasing the regenerative properties of these cells^30,37^.

**Figure 3:**
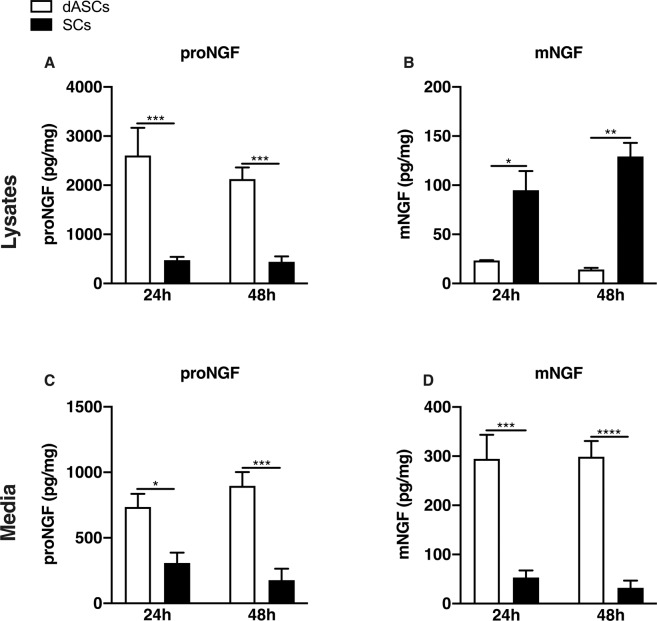
Basal levels of NGF in dASCs and in SCs. In dASCs lysates higher levels of proNGF were detected upon 24 h and 48 h of cultures than in SCs lysates (**A**, 24 h dASCs pg/mg = 2059 ± 256.8, 24 h SCs pg/mg = 473.7 ± 68.69, ***P = 0.0001; 48 h dASCs pg/mg=2124 ± 239.2, 48 h SCs pg/mg=337.4 ± 45, ***P = 0.0009; n = 3) whereas mNGF is lower than in SCs (**B**, 24 h dASCs pg/mg=23.43 ± 0.32, 24 h SCs pg/mg=94.95 ± 19.54, *P < 0.05, 48 h dASCs pg/mg=14.27 ± 1.7, 48 h SCs pg/mg=129.2 ± 13.83, **P = 0.0025; n = 3). However, dASCs release more proNGF (**C**, 24 h dASCs pg/mg=736 ± 99.76, 24 h SCs pg/mg=308 ± 78.59, *P < 0.05; 48 h dASCs pg/mg=897 ± 104.9, 48 h SCs pg/mg=176.4 ± 87.68, ***P = 0.0008; n = 3) and mNGF in the media than SCs (**D**, 24 h dASCs pg/mg=294.4 ± 49.06, 24 h SCs pg/mg=53.05 ± 14.44, ***P = 0.0001; 48 h dASCs pg/mg=299 ± 31.83, 48 h SCs pg/mg=32.2 ± 14.67, ****P < 0.0001; n = 3).

Comparing dASCs with native SCs, we showed that higher levels of proNGF were detectable, by ELISA, after 24 and 48 h of cultures in both dASCs lysates and in the supernatants (Fig. 3A,C). The baseline concentration of proNGF was 2059 ± 256.8 pg/mg in dASCs lysates, whereas in SCs we observed a mean of production of 473.7 ± 68.69 pg/mg after 24 h of culture, four times lower than dASCs (Fig. 3A, 24 h). After 48 h dASCs produced six times more than SCs, with an average of 2124 ± 239.2 pg/mg in dASCs compared to 337.4 ± 45 pg/mg in SCs (Fig. 3A, 48h). dASCs released more proNGF in the culture media with a concentration of 736 ± 99.76 pg/mg vs 308 ± 78.59 pg/mg in SCs in the first 24 h after plating (Fig. 3C, 24h). After 48h, proNGF concentration was 897 ± 104.9 pg/mg in dASCs media, five times more than SCs, where it was 176.4 ± 87.68 pg/mg (Fig. 3C, 48h).

In the lysates, mNGF concentrations were lower in dASCs at both time points analysed (Fig. 3B).

The average of concentration after 24 h was 23.43 ± 0.32 pg/mg for dASCs and an average production of 94.95 ± 19.54 pg/mg in SCs, a four-fold difference. Forty-eight hours after plating, mNGF concentration was 14.27 ± 1.7 pg/mg in dASCs whereas it was 129.2 ± 13.83 pg/mg in SCs, 9 times more than dASCs (Fig. 3B).

Finally, mNGF concentration was also more elevated in dASCs media than SCs (Fig. 3D). The concentration of mNGF was approximately 5 time higher in dASCs than SCs after 24 h (Fig. 3D, dASCs pg/mg=294.4 ± 49.06, SCs pg/mg=53.05 ± 14.44), while, after 48 h, mNGF was approximately 9 times higher in dASCs (dASCs pg/mg=299 ± 31.83, SCs pg/mg=32.2 ± 14.67).

The results obtained suggest that proNGF production and release (Fig. 3A and C), as well as NGF maturation (Fig. 3D) seem more efficient in dASCs than in SCs.

### Cholinergic modulation of proNGF and mNGF production

**Figure 4:**
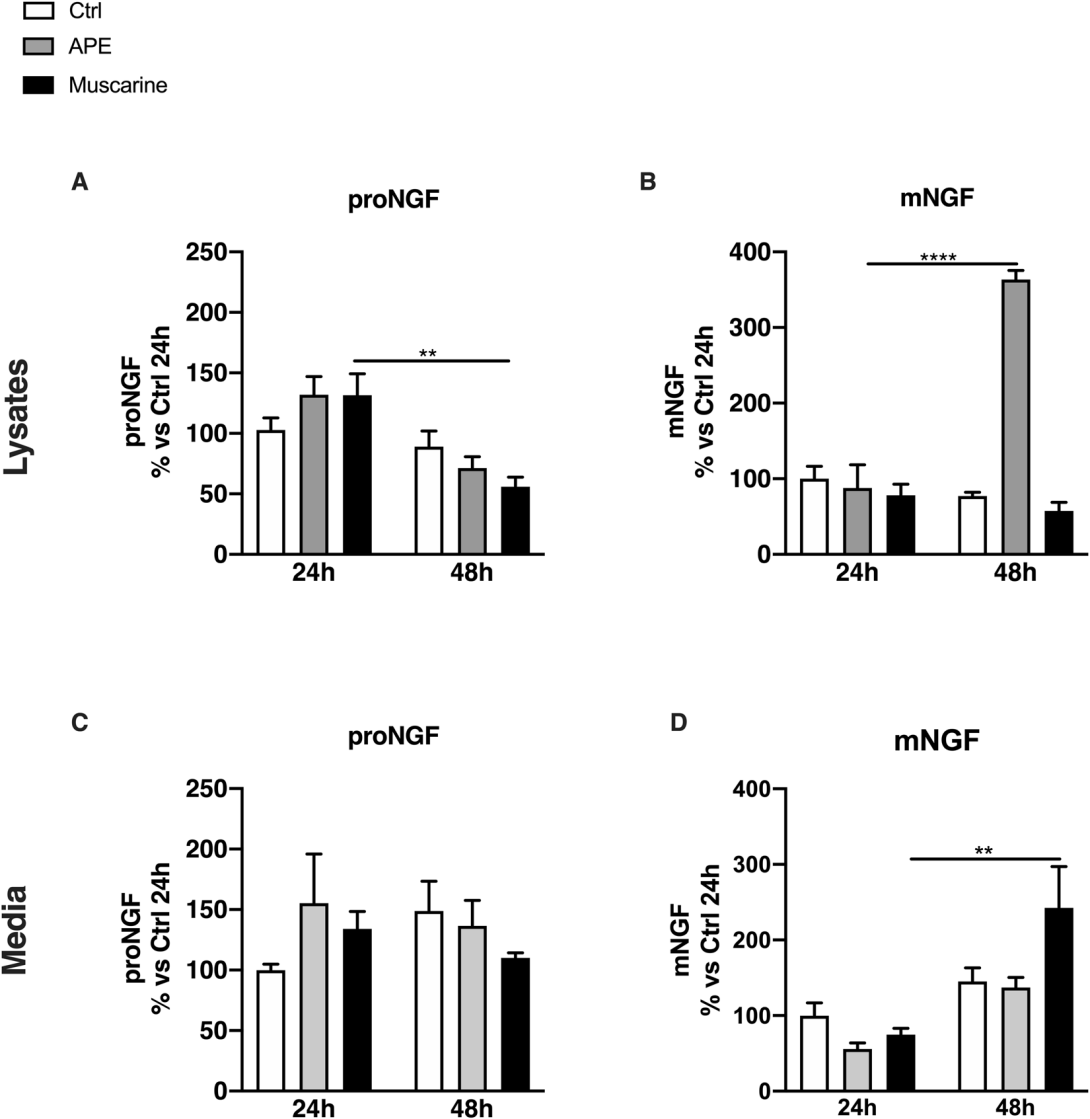
ProNGF and mNGF levels in dASCs after muscarinic receptors stimulation. **(A**) After 24 h of APE and muscarine treatment there was a faint increase of proNGF in dASCs lysates (APE: 131.9 ± 14.99% vs Ctrl, muscarine: 131.7 ± 17.61% vs Ctrl; n = 3). After 48 h there was a decrease of proNGF production (APE: 65.01 ± 9.69% vs Ctrl, muscarine: 56.57 ± 6.197% vs Ctrl, **P = 0.0091; n = 3). (**B**) Cholinergic treatments did not influence NGF maturation in the first 24 h of treatment but a strong upregulation of mNGF concentration was observed after 48 h of APE exposure (363.6 ± 11.96% vs Ctrl, ****P < 0.0001; n = 3). (**C**) Cholinergic treatments did not significantly modulate proNGF release in the cell media. (**D**) mNGF concentrations were not significantly affected by M2 selective agonist APE (24 h: 55.81 ± 7.86% vs Ctrl, 48 h:137.3 ± 13.31% vs Ctrl; n = 3) but a significant upregulation after 48 h of muscarine exposure was observed (242.5 ± 54.51% vs Ctrl, **P = 0.0023; n = 3). NGF production was normalised against the mean of control group and expressed as percentage vs. untreated controls ± SEM.

**Figure 5:**
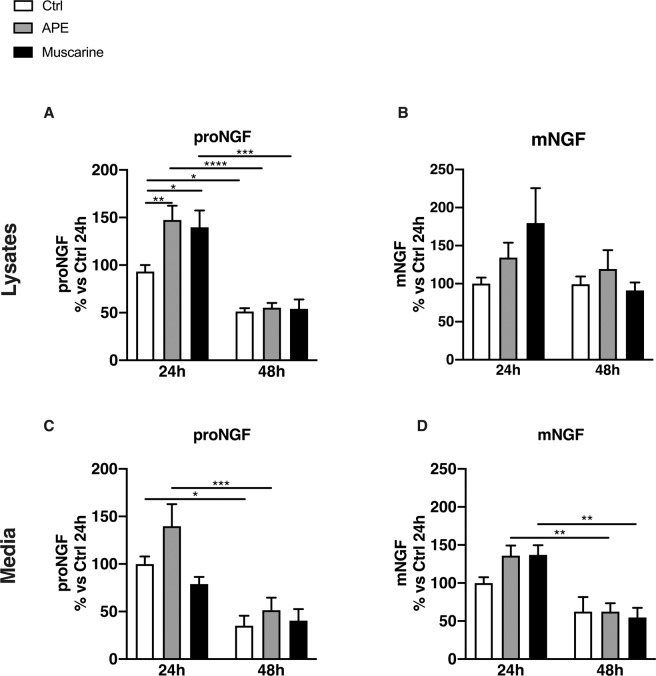
ProNGF and mNGF levels in SCs after muscarinic receptors stimulation. (**A**) proNGF basal level production significantly decreased after 48 h of culture (51.28 ± 3.45% vs Ctrl, *P < 0.05; n = 5). In SCs lysates, proNGF production increased upon 24 h of APE treatment, (147.4 ± 15.02% vs Ctrl, **P = 0.0045; n = 5) followed by decreased levels after 48 h (55.23 ± 4.96% vs Ctrl, ****P < 0.0001; n = 5). Muscarine significantly increased proNGF concentration in lysates after 24 h (139.8 ± 17.54% vs Ctrl *P < 0.05; n = 5) and decreased its effects after 48 h (54.05 ± 9.88% vs Ctrl, ***P = 0.0002; n = 5). (**B**) mNGF levels results unchanged after 24 h and 48 h of APE and muscarine exposure (24h APE:133.3 ± 16.62% vs Ctrl, 48h APE: 117±20.98% vs Ctrl, 24h muscarine: 169.4 ± 34% vs Ctrl, 48h muscarine: 102.5 ± 13.55% vs Ctrl; n = 5). (**C**) In the media there was a decrease of basal proNGF release (42.02 ± 10.64% vs Ctrl, *P < 0.05; n = 5) whereas after APE treatment there was a decrease after 48 h (51.34 ± 11.5% vs Ctrl, ***P = 0.0007; n = 5). Muscarine treatment did not exert any significant modulation on proNGF concentration in the media at both time points (24 h: 80.15 ± 5.94% vs Ctrl; 48h: 40.35 ± 9.99% vs Ctrl; n = 5). proNGF release was reduced after 48h. (**D**) APE and muscarine significantly decreased mNGF after 48 h of exposure (APE: 64.82 ±  9.6% vs Ctrl, **P = 0.0039; muscarine: 54.68 ± 10.32% vs Ctrl, **P = 0.0080; n = 5). NGF production was normalised against the mean of controls and expressed as percentage vs untreated controls ± SEM.

By ELISA assays, we evaluated the amount of proNGF and mNGF production in dASCs (Fig. 4) and SCs (Fig. 5) upon cholinergic agonists stimulation.

No significant variations in proNGF levels were observed in dASCs lysates, except for a decrease induced by muscarine after 48 h of treatment (Fig. 4A). As demonstrated in Fig. 4B, selective and non-selective cholinergic treatments did not influence NGF maturation in the first 24 h of treatment, but a significant upregulation of mNGF concentration in cell lysates was observed after 48 h of APE exposure. In dASCs, cholinergic treatments did not alter the concentration of proNGF released in the culture media (Fig. 4C). In the cell media, mNGF concentrations were not affected by muscarinic agonists in the first 24 h of stimulation, while a significant upregulation after 48 h of muscarine exposure was observed (Fig. 4D).

Using an approach similar to that followed for dASCs, NGF production, maturation and release were evaluated in native SCs (Fig. 5). proNGF basal (controls) level production significantly decreased after 48 h of cell culture (Fig. 5A). After 24 h of APE treatment, proNGF concentration was significantly increased in cell lysates (Fig. 5A), but it was decreased at control levels after 48 h (Fig. 5A). Similarly, muscarine increased proNGF concentration in lysates after 24 h of treatment, while a significant decrease was observed after 48 h (Fig. 5A). In SCs lysates, NGF maturation was unaltered after 24 h and 48h of exposure to APE and muscarine (Fig. 5B).

Similarly to what observed in cell lysates, in the culture media we observed a significant decrease of the basal proNGF released between 24 and 48 h of culture (Fig. 5C). Twenty-four hour of APE exposure increased proNGF release (Fig. 5C), while a significant decreased in concentration was observed following 48 h (Fig. 5C). There was no significant modulation of proNGF concentration in the cell media after non-selective challenge with muscarine.

Basal mNGF released in the cell media was not affected by both selective and non-selective muscarinic challenge after 24h of treatment, whereas a significant reduction was observed after 48 h in the culture media of cells treated with both muscarinic agonists (Fig. 5D).

### Cholinergic modulation of tPA and MMPs activity

**Figure 6:**
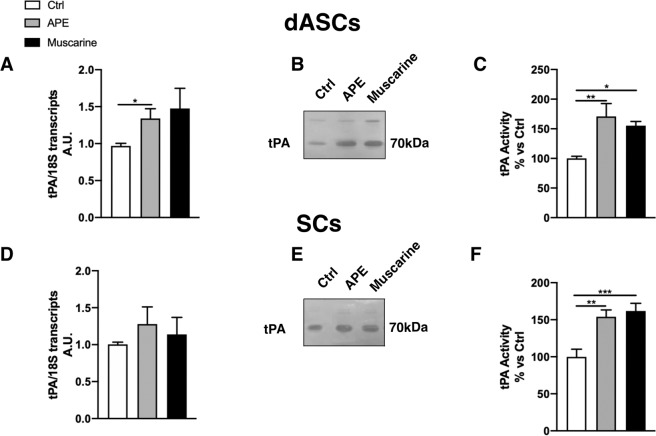
Cholinergic modulation of tPA activity. (**A**) qPCR showed an increased tPA gene expression after APE treatment in dASCs (**A**, fold change: 1.342 ± 0.13, *P < 0.05, n = 3). (**D**) tPA gene expression was not influenced by cholinergic treatments in SCs. tPA activity was significantly upregulated after APE or muscarine exposure in dASCs (**B, C**; APE: 170.9 ± 21.66% vs Ctrl, **P = 0.0028; muscarine: 155.2 ± 7.22% vs Ctrl, *P < 0.05; n = 3) and in SCs cells (**E, F**; APE: 154.1 ± 9.08% vs Ctrl, **P = 0.0025; muscarine: 161.9 ± 10.39% vs Ctrl, ***P = 0.0007; n = 3).

Tissue PA enzyme is able to promote the conversion of proNGF into mNGF^4^. In dASCs, tPA gene expression was increased after cholinergic treatments (Fig. 6A) whereas this was not observed in SCs (Fig. 6D). However, tPA activity, measured by zymography, was significantly increased in both dASCs and SCs after APE or muscarine exposure (Fig. 6).

**Figure 7:**
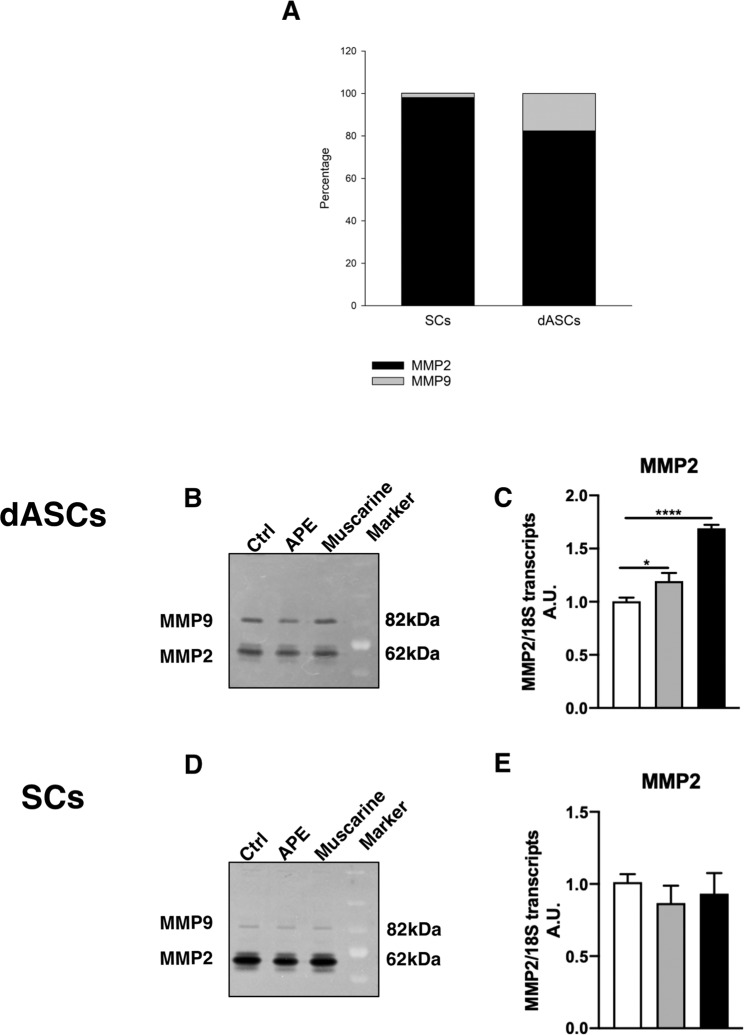
Metalloproteinases MMP2 and 9 activity in dASCs and in SCs. (**A**) MMP9 activity, responsible for NGF degradation, was significantly more active in dASCs than SCs. MMP2 and 9 activity was not influenced by cholinergic treatments in dASCs (**B**) and SCs (**D**). (**C**) MMP2 gene expression was significantly upregulated after APE and muscarine treatments in dASCs (APE fold change: 1.194 ± 0.07, *P < 0.05; muscarine fold change: 1.691 ± 0.033, ****P < 0.0001; n = 3), (**E**) whereas MMP2 gene expression was not influenced by cholinergic treatments in SCs.

Metalloproteinases, in particular MMP9, are responsible of mNGF degradation^4^ whereas MMP2 plays a relevant role in extracellular matrix remodelling, one of the event necessary to support nerve regeneration and in the modulation of pro-inflammatory cytokines (i.e. TNFα)^38^. As shown in Fig. 7A, MMP9 activity was higher in dASCs compared to SCs, whereas MMP2 activity was higher in SCs. MMP2 gene expression was significantly upregulated after APE and muscarine treatment in dASCs (Fig. 7C), but not in SCs (Fig. 7E). Conversely, MMP9 gene expression did not show significative differences in terms of transcript levels in all experimental conditions and in neither cell types (*data not shown*). However, MMP2 and 9 activity, measured by zymography, was not influenced by cholinergic treatments, remaining similar to untreated cells (Fig. 7B,D).

## Discussion

A relationship between ACh and NGF in the CNS has been previously reported. Indeed, NGF is important to regulate septo-hippocampal physiology and there are potential therapeutic uses in neurodegenerative disorders involving a cholinergic deficit^39^. Moreover, NGF was reported to attenuate cholinergic deficits in a rat model of traumatic brain injury^40^, and NGF gene transfer into aged animals can improve neuronal depolarization induced by ACh release from hippocampal synaptic terminals^41^. Conversely, ACh can regulate neurotrophin metabolism; indeed, the cholinergic system is implicated in the modulation of hippocampal NGF and BDNF mRNA expression, i.e. the injection of muscarinic agonist, pilocarpine, increases hippocampal BDNF and NGF mRNAs in postnatal and adult rats^42^.

In the present work we demonstrate for the first time that the pharmacological modulation of muscarinic receptors regulates the production and secretion of NGF also in peripheral nervous system, and in particular in SCs. Moreover, we also demonstrate the ability of dASCs to produce higher levels of NGF compared to SCs, supporting the ability of dASCs in promoting peripheral nerve regeneration.

This work follows our previous study, focused on the effects mediated by M2 muscarinic receptors in rat SC-like adipose-derived stem cells^33^; however, herein we have also analysed the effects of all other muscarinic receptors in the modulation of NGF production and release, highlighting the following points:

### Cholinergic regulation of pro-NGF isoforms

M2 selective activation with APE treatment results in a significant decrease of proNGF-B gene and protein expression in both dASCs and SCs; whilst proNGF-A gene expression is significantly downregulated in SCs and it is significantly upregulated in dASCs after both APE and muscarine treatments. Distinct proNGF isoforms are expressed in mouse tissues, resulting from alternative splicing and/or activation of different promoters^5,43,44^; as recently reported, proNGF-A has a pro-survival and differentiative effects *in vitro*^6^, whereas proNGF-B isoform acts as an apoptotic signal^7^. The longer variant proNGF-A (32–34 kDa) and the shorter variant proNGF-B (25–27 kDa) are produced in both CNS and PNS^43–46^. Our results suggest that muscarinic challenge promotes a proNGF-mediated neuroreparative response in dASCs, whilst cholinergic stimulation could improve neuronal survival and axon regeneration via proNGF-A/proNGF-B ratio modulation in glial cells.

### Effects on the expression of the low affinity p75 receptor

ProNGF preferentially binds the low-affinity p75 neurotrophic receptor (P75NTR)^47^. After APE exposure, p75NTR gene expression decreases in dASCs but remains unchanged in SCs; whilst muscarine stimulation decreases p75NTR transcript levels in dASCs but promotes its expression in SCs. Interestingly immunocytochemistry analysis demonstrates an increased expression of p75NTR protein upon both muscarinic agonists treatment but only in dASCs. Considering recent evidence suggesting that several miRNA are involved in the regulation of p75NTR in different physiological and pathological conditions^48,49^, the differential expressions of gene and protein in SCs following APE and muscarine stimulation may indicate p75NTR post-transcriptional regulation. These data, together with the reported p75NTR-mediated pro-survival effect of proNGF-A^6^, support the idea that dASCs could positively promote the neuro-reparative process after M2 receptor challenge.

### dASCs produce and release higher levels of pro- and mNGF compared to SCs

The comparison between proNGF and mNGF content in dASCs and SCs point at the former cell type as a suitable and perhaps preferable regenerative tool in the treatment of peripheral nerve lesions. To investigate proNGF release and maturation, ELISA was employed with two different antibodies to discriminate proNGF and mNGF^50^. Our data clearly indicate that dASCs produce and release higher levels of proNGF and mNGF than SCs in basal condition. In light of previous reports, demonstrating the role of NGF in the process of peripheral nerve regeneration^1,11–13^, our findings suggest that dASCs could supply a larger amount of the neurotrophic factor, if used instead of native SCs as nerve-repair promoting tool. It is worth noticing that higher levels of mNGF in dASCs may be produced both after proNGF release and proteolytic cleavage in extracellular space and after proNGF intracellular processing by furin^4^.Though this matter deserves more specific investigation, our data suggest that the extracellular conversion of proNGF into mNGF is prevalent in the dASCs phenotype.

### Cholinergic stimulation differently modulates NGF production in dASCs and SCs

All muscarinic receptor activation by muscarine should generate a balance on their effect on NGF metabolism, however it is evident that muscarine and APE treatments show similar effects, suggesting that M2 receptor may be the main subtype involved in NGF metabolism both in dASCs and SCs. The difference between the two different ligands was especially observed in mNGF levels that is higher in dASCs lysates after 48 h of APE exposure and upon 48 h of muscarine treatment in cell media. ELISA data also show a relative decrease of proNGF and mNGF concentration after 48 h of treatment, more evident in SCs. These results suggest that in SCs, despite having lower NGF levels than dASCs, muscarinic stimulation may contribute to trigger proNGF/mNGF production more efficiently. On the other hand, the higher basal levels of proNGF and mNGF observed in dASCs could explain the delayed response to muscarinic stimulation in this cell type. This hypothesis is supported by the evidence that in dASCs a decrease in proNGF followed by an increase in mNGF cellular content is observed 48 h after APE stimulation, indicating that, in these cells, the M2 stimulation promotes proNGF maturation. Conversely, the intracellular proNGF increases upon only 24 h of cholinergic treatment in SCs, suggesting that proNGF and mNGF may be previously accumulated and then released after cholinergic stimulation in SCs. Overall, these data indicate that SCs may be less reactive than dASCs in generating an NGF-based neuro-reparative process in response to muscarinic activation.

### NGF maturation and degradation machinery is differently expressed and modulated in dASCs and SCs

ProNGF can be converted to mNGF in the extracellular environment and the tissue plasminogen activator (tPA) is involved in the proNGF to mNGF cleavage^4^. The tissue plasminogen activator tPA is a key enzyme involved in several biological processes, including vascular and tissue remodelling, tumour progression^51^ and nervous system pathophysiology^52,53^. Tissue PA is a secreted protease that converts plasminogen into the active protease plasmin, which in turn participates in the cleavage of proNGF into mNGF^4^. In the PNS, tPA activity is elevated during axonal outgrowth and tPA is secreted by primary neurons and cultured SCs^54,55^, and its activity is particularly concentrated in growth cones^54^. Interestingly, the tPA system is potently induced also after peripheral nerve injury in mice^56,57^. As described above, peripheral nerve regeneration acts on the capacity of axons to regrow in a very intricate environment, composed by altered ECM, myelin and axon debris and infiltrating inflammatory cells. In these hostile conditions axonal growth seems hampered, thus regenerating neurites secrete tPA to degrade cell-cell and cell-matrix adhesion molecules. Accordingly, tPA mRNAs are strongly and early upregulated after peripheral nerve injury in Schwann cells and neurons, and their activity increases at the injury site up to 7 days post-crush^58^.

We found that tPA gene expression and activity are upregulated after 24 h of muscarinic stimulation both in dASCs and SCs. The increase of tPA activity is significant in both dASCs and SCs and this may indicate that the cholinergic stimulation may efficiently promote the proNGF maturation. In SCs, the increase of tPA activity is less evident, supporting the previous hypothesis that cholinergic stimulation in these cells promotes the proNGF and mNGF previously accumulated in the cells. In addition, tPA can convert plasminogen to plasmin, and plasmin, in turn, can work indirectly through the activation of matrix metalloproteinases (MMPs) to complement its degrading activities^59^. Accordingly, MMP9 is mainly involved in mNGF degradation^4^.

Although our data demonstrate that APE or muscarine treatments do not regulate the MMPs activity, we found a higher activity of MMP9 in dASCs than SCs. This suggests that the increased pro- and mNGF levels in dASCs supernatants indicate the possible NGF maturation and degradation in extracellular space by tPA and MMP9 activity respectively. MMP2 gene expression is significantly upregulated by cholinergic stimulation in dASCs but activity levels remain unchanged. Considering the role of MMP2 in the extracellular matrix remodelling and in the modulation of pro-inflammatory cytokines (i.e. TNFα)^38^, the presence of MMP2 activity in dASCs and in SCs may contribute to the regenerative property of these cells.

## Conclusions

Our data demonstrate that muscarinic receptors promote the NGF production and maturation in SCs and dASCs. The higher levels of basal NGF and the increase of proNGF and mNGF production upon cholinergic stimulation, accompanied by an efficient increase of tPA activity and higher levels of MMP9, suggest that dASCs are efficiently capable to produce NGF and that this production can be modulated by cholinergic stimulation. In the perspective of regenerative medicine, this feature is fundamental, suggesting the use of these cells as a reservoir of neurotrophic factors to accelerate the regenerative process. Moreover, it is known that NGF, acting through neuronal TrkA, strongly regulates myelination of DRG axons by both SCs and oligodendrocytes^60^. In accordance with our previous work on SCs^23^ and dASCs^33^, NGF release could act in an autocrine way, via trkA and p75NTR receptors, improving SCs activity during nerve regeneration. This article postulates the first evidence of the involvement of muscarinic receptors, and in particular M2 receptor, in the regulation of nerve growth factor expression and release in the PNS. Although further analyses are needed to fully elucidate the role of ACh in modulating peripheral nerve regeneration, cholinergic receptor stimulation may represent, together with dASCs, a promising and clinically applicable pharmacological intervention.

## Methods

### Statements for experiments involving the use of animals

All the experiments requiring animals were performed within the Biological Services Facilities (BSF) at the University of Manchester, in accordance with the UK Animals (Scientific Procedures) Act, 1986. Following terminal anaesthesia with CO_2_ and cervical dislocation (Schedule 1), tissues were harvested from the animals and processed as required to obtain the different cell cultures.

For experiments performed in Italy, the protocol (7FF2C.6.EXT. 96) has been approved by the Ministry of Health (Aut. N. 1184/2016-PR 16/12/2016).

### Adipose-derived stem cells isolation

ASCs were isolated from adult Sprague–Dawley rats as previously reported^15^. Visceral fat was carefully dissected and minced using a sterile razor blade. Later, tissue was enzymatically dissociated with 0.15% (w/v) collagenase type I (Invitrogen, UK) for 90 min at 37 °C under constant agitation. Collagenase was neutralized by the addition of α-Minimum Essential Medium Eagle (α-MEM, Sigma-Aldrich, Poole, UK) containing 10% (v/v) foetal bovine serum (FBS, LabTech, Uckfield, UK). Undissociated tissue was removed passing the solution through a 100μm filter and then centrifuged at 1200 rpm for 10 min. The stromal cell pellet was resuspended in α-MEM (Sigma-Aldrich, Poole, UK) containing 10% (v/v) FBS (LabTech, Uckfield, UK), 2mM L-glutamine (GE Healthcare UK, Little Chalfont, UK) and 1% (v/v) penicillin/streptomycin solution (Sigma-Aldrich, UK). Cultures were maintained at sub-confluent levels in a 37 °C incubator with 5% CO_2_.

### ASCs differentiation towards Schwann-like phenotype

ASCs were differentiated into Schwann-like cells phenotype [SC-like adipose-derived stem cells (dASCs)] using a previously established protocol^15,61^.

Sub-confluent cultures of ASCs were incubated with α-MEM (Sigma-Aldrich, Poole, UK) containing 10% FBS (LabTech, Uckfield, UK) supplemented with 1 mM β-mercaptoethanol (Sigma-Aldrich, UK) for 24 h. Cells were washed twice with HBSS (Sigma-Aldrich, UK) and new medium supplemented with 35 ng/ml all-trans-retinoic acid (Sigma-Aldrich, UK) was added for 72 h. Afterwards cells were washed twice and transferred to α-MEM (Sigma-Aldrich, Poole, UK) containing 10% FBS (LabTech, Uckfield, UK) supplemented with 5 ng/ml platelet-derived growth factor (PDGF; PeproTech Ltd., UK), 10 ng/ml basic fibroblast growth factor (bFGF; PeproTech Ltd., UK), 14 μM forskolin (Fsk; Sigma-Aldrich, UK) and 192 ng/ml glial growth factor-2 (GGF-2, Acorda, Ardsley, NY, USA) (cell differentiation medium). Cells were incubated in cell differentiation medium for 2 weeks, passaged when sub-confluent, with fresh medium added approximately every 72 h.

### Schwann cells cultures

SCs primary cultures were obtained from sciatic nerves dissected from 2-day-old Wistar pups, according to the protocol modified by Davis and Stroobant^62^. In brief, sciatic nerves were digested with trypsin/collagenase (Type I, Sigma-Aldrich, St. Louis, MA Louis, MA) and seeded into T25 flasks with fresh high glucose Dulbecco’s Modified Eagle’s Medium (DMEM, Sigma-Aldrich, Poole, UK) containing 10% foetal bovine serum (LabTech, Uckfield, UK). To selectively remove fibroblasts, cells were treated with 1 mM cytosine arabinoside (AraC, Sigma-Aldrich, UK) for 48 h and then with anti-Thy 1.1 (1:1000, Serotec, Bio-Radgroup, Hercules, CA) and rabbit complement (1:2 v/v) (Cedarlane, ON, Canada). SCs were then amplified in high glucose Dulbecco’s Modified Eagle’s Medium (DMEM, Sigma-Aldrich, Poole, UK), 10% FBS (LabTech, Uckfield, UK), 10 μM forskolin (Fsk; Sigma-Aldrich, UK) and 63 ng/ml glial growth factor-2 (GGF-2, Acorda, Ardsley, NY, USA).

SCs were incubated in 5% CO_2_ at 37 °C and maintained at sub-confluent levels onto Poly-D-Lysine (PDL, Sigma-Aldrich, UK)-coated 75 cm^2^ flasks.

### Cell treatments with cholinergic mimetics

Arecaidine Propargyl Ester hydrobromide (APE, Sigma-Aldrich, UK) is a preferring agonist of M2 receptor subtype. Its ability to selectively bind M2 muscarinic subtype has been previously demonstrated by pharmacological binding experiments and M2 knockdown in different cell culture models^22,32,63,64^. Muscarine (Sigma-Aldrich, UK) is a non-selective muscarinic receptor agonist. Both muscarinic ligands were used at the final concentration of 100μM^21–23,32,33^.

For pharmacological treatments, SCs and dASCs cultures were seeded in multi-6-well plates (Corning Life Sciences, USA) and incubated in their respective media. Once confluent, cells were rinsed and supplemented with fresh media containing 0.5% FBS (LabTech, Uckfield, UK) according to ELISA manufacturer’s protocols. After 24 h or 48 h of treatment, cells were rinsed with ice-cold phosphate buffer solution (PBS) and scraped in RIPA lysis buffer (Sigma-Aldrich, UK) containing a cocktail of protease and phosphatase inhibitors (Thermo Scientific, Loughborough, UK). After 30 min of incubation on ice and a freeze-thaw cycle, lysates were centrifuged for 20 min at 14000 rpm at 4 °C. Protein concentration was determined with the Bio-Rad detergent-compatible protein assay kit (Bio-Rad Laboratories, UK). The whole cell lysates were used for western blot analysis and ELISA of intracellular NGF as explained below. Cell media were collected and frozen until ELISA analyses. For qPCR analysis, cells were scraped with RNA cell protect reagent (Qiagen, Manchester, UK).

### Real Time PCR (qPCR)

Cells were collected at the chosen time point (24 h) and stored in RNA cell protect agent (Qiagen, Manchester, UK). Total RNA was isolated from rat SCs and dASCs using RNeasy Plus Mini Kit (Qiagen, Manchester, UK), according to the manufacturer’s protocol. Each sample was reverse-transcribed using RT^2^ First Strand Kit (Qiagen, Manchester, UK), according to the manufacturer’s protocol. Real-time PCR was performed with RT^2^ SYBR Green qPCR Mastermix (Qiagen, Manchester, UK) using Corbett Rotor Gene 6000 real-time cycler (Qiagen, UK). All reactions were carried out in triplicate and the following protocol used: hot start for 10 min at 95 °C, followed by 45 cycles of 15 sec at 95 °C, annealing for 30 sec at 55 °C and extension for 30 sec at 72 °C. The sequences of the used primers are reported in Table 1. Data were normalized with 18 S housekeeping gene and the ΔΔCt method was used to determine the fold changes in the gene expression, as compared to control.

**Table 1:** List of qPCR primers.

Primer	Forward (5′-3′)	Reverse (5′-3′)
NGF	AAGGACGCAGCTTTCTATCC	CTATCTGTGTACGGTTCTGCC
proNGF-A	GTGTCCACCCATCTGCTAGG	CACTGAGGTGAGCTTGGGTC
proNGF-B	CCTGGAGCCGAAGGGGAG	CACTGAGGTGAGCTTGGGTC
p75NTR	CATCTCTGTGGACAGCCAGA	CTCTACCTCCTCACGCTTGG
tPA	AGCTAATCAGCTCAGCGCCAA	CTCCCCGTTTCTTCCGTGTC
MMP2	CAGGGAATGAGTACTGGGTCTATT	ACTCCAGTTAAAGGCAGCGTCTAC
18S	GGATCCATTGGAGGGCAAGT	ACGAGCTTTTTAACTGCAGCAA

### Immunocytochemistry

After fixation with 4% Paraformaldehyde (PFA) for 20 min at RT, cells were incubated with 0.2% TritonX-100 for 30 min at RT. Then cells were washed twice with phosphate buffer Saline (PBS) and treated with block solution (PBS 0.1% TritonX-100 and 10% normal donkey serum (NDS)) for 1 h at RT. The next step was an incubation with primary antibody, rabbit anti-p75 NGF receptor antibody (Abcam, UK) at 4 °C overnight. The day after, cells were washed with PBS three times for 10 min and they were incubated with secondary antibody (Donkey anti-rabbit IgG (H + L) Highly Cross-Adsorbed Secondary Antibody, Alexa Fluor 568, Life Technologies, UK) in 0.1% TritonX-100, 0.1% (w/v) BSA, 0.1% (w/v) Sodium Azide in PBS for 1 h at RT. Afterwards, plates were washed 3 times with PBS and slides were mounted with Vectashield mounting medium for fluorescence containing 4′-6′-diamidino-2-phenylindole for nuclear staining (H1200, Vector Lab, DBA, Milan, Italy). Images were taken using a fluorescence microscope (Olympus IX51, Southend-on-Sea, UK) and processed with ImageJ 64 imaging software (National Institutes of Health, NIH, 469 Bethesda, MD, USA).

### Protein extraction and Western blot

Cells were homogenized in ice-cold lysis buffer (20 mM Hepes pH 7.9; 150 mM sodium chloride; 1% Nonidet P-40; 0.1 mM EDTA; 0.1 mM EGTA). Total protein content was evaluated by DCTM Protein Assay (Biorad, Italy). Twenty micrograms of proteins were resolved in 8–12% SDS-PAGE, blotted onto nitrocellulose membrane and processed for Western blot according to what has been previously reported^50^. Used antibodies are detailed in Table 2. Densitometry was performed on scanned immunoblot images using ImageJ gel analysis tool (National Institutes of Health, NIH, 469 Bethesda, MD, USA). The optical density (OD) of each protein band was normalized against the OD of the β-actin band. The results from at least three independent experiments were averaged and the standard error of the mean (SEM) was calculated.

**Table 2:** List of antibodies.

Primary antibody (catalog, manufacture)	Application/Dilution	Secondary antibody (catalog, manufacture, dilution)
rabbit **anti-NGF M20** (sc-549, Santa Cruz)	WB: 1:1000	**HRP-linked anti-rabbit IgG** 7074, Cell Signaling 1:4000
mouse **anti-β-actin** (A5441, Sigma-Aldrich)	WB: 1:10000	**HRP-linked anti-mouse IgG** 7076, Cell Signaling 1:5000
rabbit **anti-proNGF** (EP1318Y, Abcam 68151)	Detection ELISA: 1:5000	**HRP-linked anti-rabbit IgG** 7074, Cell Signaling 1:1000
goat **anti-NGF** (AF-556-NA, R&D)	Capture ELISA: 0.4 μg/ml	
mouse **anti-NGF 27/21** (52602, Millipore)	Detection ELISA: 1:1000	**HRP-linked anti-mouse IgG** 7076, Cell Signaling 1:1000
rabbit **anti-p75NTR** (Abcam, UK)	ICC: 1:100	Donkey **anti-rabbit IgG** (H + L) Highly Cross-Adsorbed Secondary Antibody, **Alexa Fluor 568** Life Technologies 1:500

### Enzyme-linked immunosorbent assay (ELISA)

proNGF and mNGF content in cell lysates and conditioned media was measured by specific ELISA, as previously described^50^. Briefly, capture antibody was the same for both the ELISA assays (Table 2) and was incubated overnight at room temperature (RT). Unbound antibody was removed by washing the plate once with washing buffer (0.5% (v/v) Tween-20 in PBS). After blocking 1 h at RT with 1% (w/v) BSA in PBS, the plate was rinsed with washing buffer and samples or standard curves were added to the wells and incubated for 2 h at RT. The microwells were then rinsed three times and incubated with specific detection antibodies dissolved in blocking buffer for 2 h, at RT. After three washes to remove unbound detection antibody, HRP-conjugated antibody, diluted in blocking buffer, was added and incubated for 1 h at RT. To visualize antibody reactivity, the chromogenic substrate 3’,3’,5’,5’-tetramethylbenzidine (TMB, cat. T8768, Sigma-Aldrich, UK) was used and colour development was stopped by adding 1 N HCl. The colorimetric reaction was measured in absorbance mode at 450 nm by a Multiskan EX ELISA reader (Thermo Fisher Scientific Laboratory).

### Zymography for matrix metalloproteinases (MMPs) detection and analysis of PAs activities

Gelatinolytic activity of conditioned media was assayed as previously described^65^. Aliquots of conditioned media were analysed on 7% sodium dodecyl sulfate polyacrylamide gel electrophoresis SDS-PAGE containing 0.1% gelatin under non-reducing conditions. Following electrophoresis, gels were washed twice in 2.5% TritonX-100 for 30 min at RT to remove SDS and then in water for 30 minutes. The gels were incubated at 37 °C overnight in substrate buffer, stained with 0.5% Coomassie Brilliant Blue R250 and distained in 30% methanol and 10% glacial acetic acid (vol⁄vol). To analyse PA activity, aliquots of conditioned media were separated by 10% SDS-PAGE under non-reducing conditions^66^. After electrophoresis, gel was washed in 2.5% TritonX-100 for 30 minutes at RT to remove SDS and then in water for 30 minutes. The TritonX-100 washed gel was placed on a casein-agar-plasminogen underlay, as previously described^67^. All the bands were plasminogen dependent. The gels were photographed and the densitometric analysis was performed using ImageJ software (National Institutes of Health, NIH, 469 Bethesda, MD, USA) to obtain semiquantitative estimation of protease activities. The values were normalized to protein content. Molecular weights were calculated from the position of pre-stained molecular weight markers subjected to electrophoresis in parallel lanes.

### Data analysis

Data analyses were performed with GraphPad Prism (ver 7.0, GraphPad Software Inc, La Jolla, CA, USA). Data were presented as the mean ± standard error of the mean (SEM). Student’s t-test or one-way ANOVA analyses with Bonferroni’ or Tukey’s post-tests were used in Western Blotting and qPCR analyses. Statistical significance for the ELISA experiments were estimated by two-way ANOVA with Tukey’s post-tests. A value of p < 0.05 was considered statistically significant: p < 0.05(*), p < 0.01(**), and p < 0.001(***) p < 0.0001(****). The densitometric analysis of the RT-qPCR, Immunocytochemistry and Western blots were measured by ImageJ software (National Institutes of Health, NIH, 469 Bethesda, MD, USA).
